# Optimizing the delivery of chemotherapy in the setting of immunotherapy in a preclinical glioblastoma model

**DOI:** 10.1186/2051-1426-3-S2-P307

**Published:** 2015-11-04

**Authors:** Dimitrios Mathios, Jennifer Kim, Jillian Phallen, Antonella Mangraviti, Chul-Kee Park, Debebe Theodros, Christopher Jackson, Tomas Garzon-Muvdi, Eileen Kim, Xiaobu Ye, Betty Tyler, Henry Brem, Drew Pardoll, Michael Lim

**Affiliations:** 1 Johns Hopkins University, Baltimore, MD, USA; 2 Seoul National University, Seoul, Korea, Republic Of; 3 The Johns Hopkins University Department of Neurosurgery, Baltimore, MD, USA; 4 Johns Hopkins University School of Medicine, Baltimore, MD, USA

## Background

Glioblastoma, despite the use of radiation and chemotherapy, remains vastly lethal. Cancer immunotherapy has entered the era of maturity with new compounds like anti-PD-1 FDA approved to treat advanced tumors. However, the use of immunotherapy in the setting of chemotherapy presents challenges, as chemotherapy causes lymphodepletion and potentially antagonizes immunotherapy. In this work, we studied ways to optimize the delivery of chemotherapy in the setting of immunotherapy.

## Methods

Using the GL-261 mouse glioblastoma model, we carried out a series of survival, immunophenotypic and mechanistic experiments to identify the optimal timing and delivery method of chemotherapy (BCNU, temozolomide) in relation to immunotherapy (anti-PD-1).

## Results

Local chemotherapy (LC) in the form of chemotherapy eluted polymer combined with anti-PD-1 monoclonal antibody led to superior survival and tumor eradication compared to monotherapies and the combination of anti-PD-1 with systemic chemotherapy. The use of systemic chemotherapy abrogated the survival benefit anti-PD-1 produced as a monotherapy in a dose dependent manner. Furthermore, systemic chemotherapy caused lymphopenia and decreased T cell effector function in a dose dependent fashion, while the combination of LC and anti-PD-1 resulted in the highest immune activation. LC increased the infiltration of dendritic cells intratumorally. Utilizing the antigen specific ova system we showed that LC allowed for expansion of antigen specific T cells while systemic chemotherapy thwarted the homing and expansion of these cells. We additionally found that systemic chemotherapy abrogated the creation of long-term memory that anti-PD-1 alone created. Mice treated with anti-PD-1 and systemic chemotherapy and rechallenged with GL-261 tumors failed to reject the tumor due to the persistent lymphodepletion caused by systemic chemotherapy as well as functional impairment of T memory cells. However, LC in combination with anti-PD1 preserved a long-term memory response. We further studied the long-term effects of SC in immunotherapy; mice treated with SC for 2 weeks were given an extended chemotherapy break (2 months) to allow the immune system to rest and were subsequently treated with anti-PD-1. Surprisingly, these mice failed to show the same survival benefit anti-PD-1 only treated mice exhibited.

## Conclusions

LC in combination with anti-PD-1 creates a robust antitumor immune response, increases antigen presentation and expands tumor specific T cells resulting in the cure of well established tumors in the majority of mice, while at the same time preserves a long-term memory antitumor response.

